# Tight Coupling of *Glaciecola* spp. and Diatoms during Cold-Water Phytoplankton Spring Blooms

**DOI:** 10.3389/fmicb.2017.00027

**Published:** 2017-01-19

**Authors:** Markus von Scheibner, Ulrich Sommer, Klaus Jürgens

**Affiliations:** ^1^Leibniz Institute for Baltic Sea Research WarnemündeRostock, Germany; ^2^Helmholtz Centre for Ocean ResearchKiel, Germany

**Keywords:** *Glaciecola*, phytoplankton, spring bloom, marine bacteria, temperature, Baltic Sea, CARD-FISH

## Abstract

Early spring phytoplankton blooms can occur at very low water temperatures but they are often decoupled from bacterial growth, which is assumed to be often temperature controlled. In a previous mesocosm study with Baltic Sea plankton communities, an early diatom bloom was associated with a high relative abundance of *Glaciecola* sequences (*Gammaproteobacteria*), at both low (2°C) and elevated (8°C) temperatures, suggesting an important role for this genus in phytoplankton-bacteria coupling. In this study, the temperature-dependent dynamics of free-living *Glaciecola* spp. during the bloom were analyzed by catalyzed reporter deposition fluorescence *in situ* hybridization using a newly developed probe. The analysis revealed the appearance of *Glaciecola* spp. in this and in previous spring mesocosm experiments as the dominating bacterial clade during diatom blooms, with a close coupling between the population dynamics of *Glaciecola* and phytoplankton development. Although elevated temperature resulted in a higher abundance and a higher net growth rate of *Glaciecola* spp. (Q_10_ ∼ 2.2), their growth was, in contrast to that of the bulk bacterial assemblages, not suppressed at 2°C and showed a similar pattern at 8°C. Independent of temperature, the highest abundance of *Glaciecola* spp. (24.0 ± 10.0% of total cell number) occurred during the peak of the phytoplankton bloom. Together with the slightly larger cell size of *Glaciecola*, this resulted in a ∼30% contribution of *Glaciecola* to total bacterial biomass. Overall, the results of this and previous studies suggest that *Glaciecola* has an ecological niche during early diatom blooms at low temperatures, when it becomes a dominant consumer of phytoplankton-derived dissolved organic matter.

## Introduction

The predicted increase in surface temperatures of 2–5° by the end of this century (IPCC, 2013) will lead to warmer oceans (Levitus, 2000), with important implications for pelagic communities and biotic interactions (Petchey et al., 1999; Burrows et al., 2011). For example, the differential impact of warming on autotrophic production and the consumption of dissolved organic matter (DOM) by heterotrophs induces a higher rate of bacterial degradation of phytoplankton-derived organic matter (Wohlers et al., 2009). At low temperatures, phytoplankton spring blooms can be temporally decoupled from bacterial degradation (Pomeroy and Deibel, 1986; Lignell et al., 1993; Bird and Karl, 1999), whereas rising water temperatures intensify phytoplankton-bacteria coupling and thereby enhance carbon flow into the microbial food web (Müren et al., 2005; Morán et al., 2006; Hoppe et al., 2008; Degerman et al., 2013). The mechanisms underlying these processes are poorly understood, although they probably depend on the ability of the bacterial assemblage to respond rapidly to the input of labile dissolved organic carbon (DOC) supplied by blooming phytoplankton (Pinhassi et al., 2004; Grossart et al., 2005; Sapp et al., 2007; Teeling et al., 2012). This has indeed been observed for bacteria belonging to *Alteromonadales* (*Gammaproteobacteria*), in which both cell abundance and transcriptional activities increase rapidly in response to DOC, particularly phytoplankton-derived organic carbon (Eilers et al., 2000; McCarren et al., 2010; Sarmento and Gasol, 2012; Beier et al., 2015). Although *Alteromonadales* are strongly grazed by bacterivorous protists (e.g., Beardsley et al., 2003), high proportional abundances have nonetheless been detected during marine phytoplankton spring blooms (Tada et al., 2011; Teeling et al., 2012), indicating the central role of these bacteria in the utilization of phytoplankton-derived DOC (Pedler et al., 2014).

The succession of dominant, free-living bacterial lineages during the proliferation of a phytoplankton spring bloom has not been well-studied at high taxonomic resolution, with a few exceptions (Teeling et al., 2012). Most studies quantitatively examined the bacterial succession during the phytoplankton blooms, using relatively broad phylogenetic probes such as those for *Bacteroidetes*, *Gammaproteobacteria*, and *Roseobacte*r in marine environments (Alderkamp et al., 2006; Tada et al., 2011; Sintes et al., 2013) and for *Actinobacteria*, *Bacteroidetes*, and *Betaproteobacteria* in limnic systems (Zeder et al., 2009; Eckert et al., 2012). To our knowledge, no study has quantified the species- or genus-level dynamics of the dominant bacteria during the development of a phytoplankton spring bloom. The identification of these bacterial taxa would enable a more detailed investigation of their role during early phytoplankton spring blooms, when low water temperatures are thought to otherwise suppress most bacterial activities (Pomeroy and Deibel, 1986).

Mesocosm studies have shown that warming results in a tighter phytoplankton-bacterial coupling and an enhanced carbon flow into the microbial food web (Hoppe et al., 2008; Wohlers et al., 2009; Wohlers-Zöllner et al., 2012; von Scheibner et al., 2014). Reduced bacterial development in the colder treatments is due to a combination of lower growth rates and relatively temperature-insensitive protist grazing pressure (von Scheibner et al., 2014). In this study, we were particularly interested in following the growth dynamics of *Glaciecola* spp. (*Alteromonadaceae*, *Gammaproteobacteria*), identified as a dominant taxa in a previous mesocosm study carried out during early phytoplankton bloom conditions. We also examined how the growth of these bacteria is affected by an increase in temperature. The population dynamics of *Glaciecola* spp., were investigated by using a newly developed, specific catalyzed reporter deposition fluorescence *in situ* hybridization (CARD-FISH) probe to analyze the experimental phytoplankton blooms that developed at low (*in situ*: ∼2°C) and high (Δ+6°C ∼8°C) temperatures in the mesocosm experiment reported in von Scheibner et al. (2014). The results showed that *Glaciecola* spp. dominated the free-living bacterial assemblages in the mesocosms, despite intense grazing pressure, and thus presumably play an important role in carbon processing during diatom blooms, both at low and at elevated temperatures.

## Materials and Methods

### Experimental Design and Sampling

The experiment was performed within the priority program AQUASHIFT (Sommer et al., 2007), using 12 indoor mesocosms maintained in four climate chambers at the GEOMAR (Kiel, Germany) from 6 February to 26 March 2008, as previously described in detail with respect to the phytoplankton development and experimental setup (Lewandowska and Sommer, 2010), biogeochemistry (Biermann et al., 2014), and microbial response (von Scheibner et al., 2014). Briefly, the 12 mesocosms, each with a volume of 1400 L, were filled simultaneously with unfiltered brackish seawater from the Kiel Fjord (Baltic Sea) and thus contained the natural winter/spring community (*in situ* temperature of 4.1°C). They were then incubated at either ∼2.0°C (= Δ0°C; low temperature) or ∼8.0°C (= Δ6°C; high temperature). The experiment also cross-linked the two temperature scenarios with three different light conditions. However, since the latter had no significant effect on either phytoplankton or bacterial parameters, the respective mesocosms were pooled such that six replicate mesocosms were available for each temperature (Lewandowska and Sommer, 2010; von Scheibner et al., 2014).

Samples used to determine bacterial abundance were fixed with 2% (final concentration) formaldehyde, filtered onto 0.2-μm polycarbonate filters (Whatman), stained with 4′,6-diamidino-2-phenylindole (DAPI), and then counted on a Zeiss Axioplan epifluorescence microscope at 1000× magnification. For bacterial biomass estimations, the area of the DAPI-stained bacterial cells was measured with the program Cell-IP (Olympus). The results were projected to cell volume by assuming a cylindrical shape for the cells and then transforming that value into biomass using the equation: fg C cell^-1^ = 133.754 × V^0.438^, according to Romanova and Sazhin (2010).

For the enumeration of heterotrophic nanoflagellates (HNFs), the samples were filtered onto 0.8-μm black polycarbonate filters (Whatman), stained with DAPI, and counted using an epifluorescence microscope (Axioskop2 mote plus, Zeiss). Biovolumes were calculated from the mean cell diameter and converted to carbon using a conversion factor of 220 fg C cm^3^ (for details, see von Scheibner et al., 2014).

The *Glaciecola* probe was also used to examine samples from a AQUASHIFT mesocosm experiment performed at the GEOMAR in 2006. The experimental setup was similar and the temperature range was the same (Δ0°C to Δ+6°C in four temperature steps). However, the duplicate mesocosms were exposed to only one light condition (Sommer et al., 2007). The 0.2-μm polycarbonate filters (3.0-μm prefiltered) obtained in that study were stored at -80°C. For the purposes of the present work, we selected filters representing each of the eight mesocosms at the chlorophyll-*a* (Chl *a*) peak of the diatom-dominated phytoplankton bloom.

To assess the *in situ* abundance of *Glaciecola* spp. during early spring bloom conditions in the Baltic Sea using the CARD-FISH probe, eight surface samples were collected during a research cruise with the *R/V Alkor* between 4 and 11 March 2009. Water samples (30–50 ml) obtained from two stations in the Gulf of Finland, two stations in the central Baltic Sea (Gotland Basin), and four stations in the southern Baltic Sea (near the coast of Germany) were filtered onto 0.2-μm polycarbonate filters and stored first at -50°C on the ship and then later, in the institute, at -80°C, until processing.

### CARD-FISH and Evaluation of the GC1252 Probe

In a previous mesocosm experiment designed to study a phytoplankton spring bloom at low temperatures, nearly full-length 16S rRNA gene sequences were retrieved from clone libraries (von Scheibner et al., 2014). One of the dominant sequences occurring during the peak of the bloom belonged to the bacterial genus *Glaciecola*.

A CARD-FISH probe highly specific for the *Glaciecola* operational taxonomic unit (OTU) was designed using the probe design tool in the ARB program suite (Ludwig et al., 2004). The resulting probe, GC1252 (5′-AGGGATGCAAACTGGTGACAGT-3′), had five outgroup hits against *Glaciecola* and 30 hits within *Glaciecola* according to SILVA TestProbe 3.0, effective Jan. 2016 (**Supplementary Table S2**). It was named GC1252 in reference to *Glaciecola* (GC) and the banding position with respect to the 16S RNA of *Escherichia coli* (1252). A recent study revealed that the genus *Glaciecola* comprises *Glaciecola* and *Paraglaciecola* (Shivaji and Reddy, 2014), but probe GC1252 targets a small clade within the genus *Glaciecola*, including the cultivated strains *Glaciecola pallidula* and *Glaciecola nitratireducens* and one uncultured Antarctic sea ice bacterium within *Paraglaciecola* (**Supplementary Table S1**). Probe GC1252 was validated using a *Glaciecola pallidula* culture (Bowman et al., 1998) as a positive control and *Alteromonas macleodii* (Baumann et al., 1972), *Colwellia psychrerythraea* (Deming et al., 1988), and *Escherichia coli* (Migula, 1895) cultures as negative controls. To determine the optimal formamide concentration in the hybridization buffer, hybridizations were performed within a formamide concentration range of 0–60% using 5% increments. The optimal formamide concentration for the detection of the *Glaciecola* clade was 55%; at this concentration a positive signal was obtained only with the positive control strain *Glaciecola pallidula*. The phylogenetic tree of the genus *Glaciecola* was constructed based on the full-length 16S rRNA sequences of selected strains within the SILVA database SSU_NR_123 (Quast et al., 2013) using the maximum-likelihood analysis (PHYML, DNA) provided in the ARB 6 program suite (Ludwig et al., 2004) and including clonal sequences determined in the mesocosm experiment (von Scheibner et al., 2014). *Colwellia psychrerythraea* served as the outgroup taxa.

The enumeration of *Glaciecola* sp. and other free-living bacteria by CARD-FISH was based on samples previously filtered onto 0.2-μm polycarbonate filters (3.0-μm prefiltered), as described in detail in von Scheibner et al. (2014). CARD-FISH was performed according to a slightly modified version of the protocol of Pernthaler et al. (2002). Briefly, the samples were hybridized at 35°C for at least 2 h or overnight with the following horseradish-peroxidase-labeled probes (50 pmol μl^-1^, biomers.net, Germany) prepared in hybridization buffer (1:150) containing 55% formamide: EUB338 I-III (Daims et al., 1999), GAM42a + GAM42a-C (Manz et al., 1992), GC1252 (this study), and, as the negative control, non-EUB (Wallner et al., 1993) (**Table 1**). The hybridized cells were counted by automated image acquisition using an epifluorescence microscope in combination with a Colibri LED unit (Axioskop2 mote plus, Zeiss, Germany) according to Zeder et al. (2009). The acquired images were quality-controlled using the software AIQC, developed by Zeder et al. (2009). The images were then manually checked; only samples with at least 1000 positive DAPI counts were used for further analysis.

**Table 1:** Overview of the CARD-FISH (catalyzed reporter deposition fluorescence *in situ* hybridization) probes used in this study.

Probe	Target	Sequence (5′–3′)	Formamide (%)	Reference
EUB338 I-III	Most *Bacteria*	GCTGCCTCCCGTAGGAGTGCAGCCACCCGTAGGTGTGCTGCCACCCGTAGGTGT	55	Daims et al., 1999
GAM42a	*Gammaproteobacteria*	GCCTTCCCACATCGTTT	55	Manz et al., 1992
GAM42a-C	Competitor for GAM42a	AGGGAUGCAAACUGGUGACAGU	55	Manz et al., 1992
GC1252	*Glaciecola* clade	AGGGATGCAAACTGGTGACAGT	55	This study
Non-EUB	Negative control	ACTCCTACGGGAGGCAGC	55	Wallner et al., 1993


Additionally, we combined microautoradiography with CARD-FISH (micro-CARD-FISH) to evaluate the activity of the *Glaciecola* clade based on its incorporation of [^3^H]-leucine, following the protocol described in Teira et al. (2004). In brief, 4 ml of unfiltered water collected from each mesocosm during the phytoplankton bloom peak was incubated with [^3^H]-leucine (20 nM final concentration) at the *in situ* temperature for 2 h. Incubations were terminated by the addition of formaldehyde (2% final concentration). All samples were filtered onto 0.2-μm polycarbonate filters (Millipore, Darmstadt, Germany) softly bedded on cellulose acetate filters (Millipore, 0.45 μm), rinsed with Milli-Q water, dried, and stored at -20°C until further processing for microautoradiography as described in Teira et al. (2004).

### Calculation of Growth Rates and Statistical Analysis

Both the net growth rates of the individual bacterial groups detected by the CARD-FISH probes and the total cell numbers were calculated with respect to the changes in cell abundance over time. Net growth rates were calculated according to Peters (2002), assuming exponential growth. The growth rates during the period of increasing cell abundance, defined as between the start (day 8) and peak (day 18) of the phytoplankton bloom, were calculated separately for each mesocosm (six replicate mesocosms per temperature). This period covered five time points for the determination of total bacterial abundance and at least four time points for the detection of *Glaciecola* (GC1252), *Gammaproteobacteria* (Gam 42a), and total Bacteria (EUBI-III). These net growth rates can be regarded as minimum estimates and would probably be higher with more frequent sampling. However, the major bacterial increase that occurred in parallel with bloom development was similarly covered at the low and high temperatures, which therefore enabled comparisons of the results. The temperature sensitivity of a given process can be described by its Q_10_ value, which is the factorial increase in the rate of that process in response to an increase in temperature of 10°C. The Q_10_ value was calculated for the increase in cell abundance of the different bacterial groups, and the significance of the temperature effects was evaluated using an independent sample *t*-test.

## Results

**FIGURE 1:**
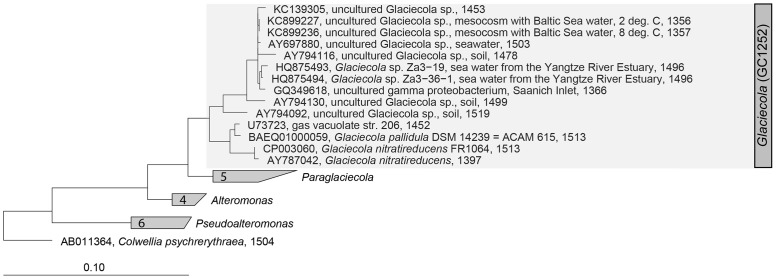
**Maximum likelihood (PHYML, DNA) tree of the genus *Glaciecola* within the family *Alteromonadaceae* (*Gammaproteobacteria*).** The specific, newly developed CARD-FISH (catalyzed reporter deposition fluorescence *in situ* hybridization) probe GC1252 targets a narrow clade within the genus *Glaciecola* (shaded in gray).

During the peak of the phytoplankton bloom, bacterial community composition, analyzed by 16S rRNA Sanger-sequenced clone libraries, was clearly dominated by *Gammaproteobacteria* under the low (∼2.0°C) and high (∼8.0°C) temperature regimes, as previously reported (von Scheibner et al., 2014). The most abundant OTU was that of a *Glaciecola* sp. closely related to *Glaciecola* sp. HTCC2999 (98%) and to *Glaciecola pallidula* (96%), which accounted for 57% and 44% of the total clones in the colder and warmer treatments, respectively (von Scheibner et al., 2014). A precise cellular quantification of *Glaciecola* spp. using the newly designed probe GC1252 was now possible. However, while GC1252 was primarily designed to detect the abundant *Glaciecola* sequence, it is also able to target a narrow clade containing 30 taxa within the genus *Glaciecola* (**Figure 1**; **Supplementary Tables S1** and **S2**), including two OTUs previously found in the clone libraries (von Scheibner et al., 2014). In our mesocosm experiment, GC1252-positive cells were present as uniform free-living, relatively large rod-shaped cells (**Supplementary Figure S1**). The measured area of *Glaciecola* spp. cells was on average 34% larger than that of all bacterial cells, resulting in a ∼43% higher biomass per cell.

**FIGURE 2:**
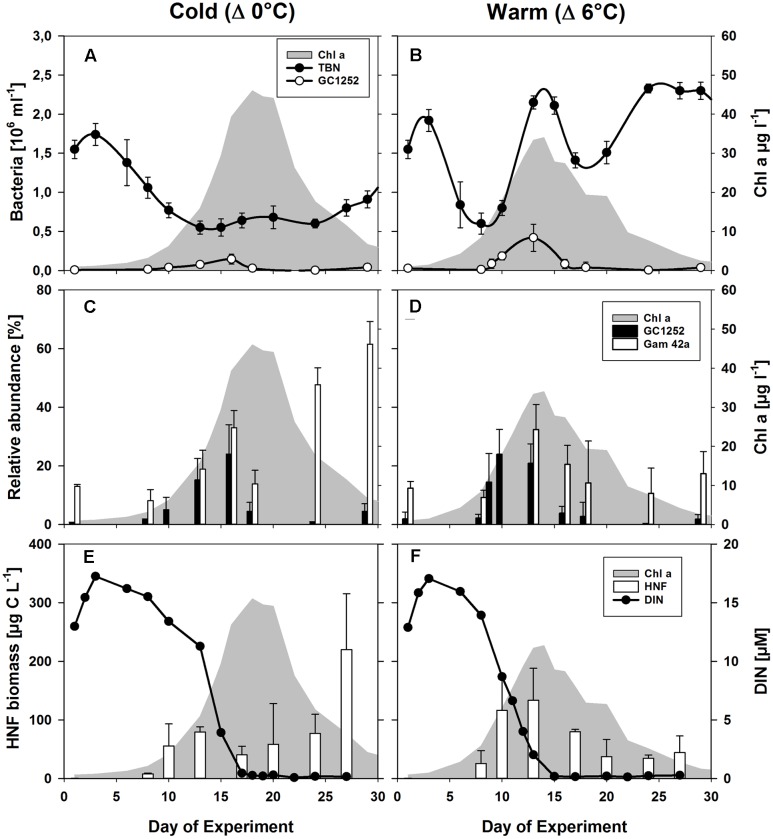
**Phytoplankton and bacterial development during the mesocosm experiment.** **(A,B)** Changes in the abundances of total bacterial numbers (TBN) and of cells detectable by CARD-FISH using the probes GC1252 (*Glaciecola*) during the phytoplankton blooms (shaded in gray) for the low (Δ0°C ∼ 2°C) and high (Δ6°C ∼ 8°C) temperature scenarios. **(C,D)** Relative abundance of cells detectable by FISH using the probes Gam42a (*Gammaproteobacteria*) and GC1252 (*Glaciecola*). **(E,F)** Estimated heterotrophic nanoflagellate (HNF) biomass (von Scheibner et al., 2014) and dissolved inorganic nitrogen (DIN). From Biermann et al. (2014).

Phytoplankton spring blooms with similar growth patterns developed in all 12 mesocosms, but significant differences were consistently observed between the two temperature scenarios (Δ0°C and Δ+6°C). These included the lower Chl *a* maximum and its advanced onset (4–6 days) with warming (**Figure 2**) (Lewandowska and Sommer, 2010; Biermann et al., 2014). The phytoplankton bloom was dominated by diatoms, with *Skeletonema costatum* as the dominant species under warmer conditions (55 ± 8% of total phytoplankton biomass vs. 19 ± 5% under colder conditions) and *Thalassiosira rotula* as the most abundant species under colder conditions (39 ± 6% of total phytoplankton biomass vs. to 3 ± 1% under warmer conditions) (Lewandowska and Sommer, 2010). Bacterial abundance was strongly enhanced during the phytoplankton bloom at the higher temperature, reaching 2.15 ± 0.08 × 10^6^ cells ml^-1^ at its peak (**Figure 2B**) compared to 0.64 ± 0.10 × 10^6^ cells ml^-1^ in the colder treatment (**Figure 2A**).

The population dynamics of *Glaciecola* spp. (GC1252-positive cells), especially during the exponential growth phase, were followed by analyzing samples from all 12 mesocosms over the entire course of the phytoplankton spring bloom (**Figure 2**). *Glaciecola* spp. were of low abundance before the bloom, constituting only 1–2% of the total free-living bacterial cells (DAPI-stained cells), except in mesocosm IV (5.7%). A strong increase in *Glaciecola* spp. was first observed in parallel with the developing phytoplankton bloom, although it was somewhat slower in the colder treatments (**Figure 2**). In the warmer treatments, the relative abundance of *Glaciecola* spp. developed in parallel with that of the whole bacterial assemblage, increasing within 2 days from 2.2 ± 1.3% (0.13 ± 0.08 10^5^ cells ml^-1^) on day 8 to 24.0 ± 8.5% (1.85 ± 0.51 10^5^ cells ml^-1^) on day 10 (**Figure 2**). In the colder treatments, *Glaciecola* spp. increased even though total bacterial numbers stayed constant or in some cases decreased. The observed increase occurred over 5 days, from 1.5 ± 0.4% (0.16 ± 0.04 10^5^ cells ml^-1^) on day 8 to 15.2 ± 7.3% (0.77 ± 0.31 10^5^ cells ml^-1^) on day 13 (**Figure 2**).

The resulting doubling times of *Glaciecola* spp., calculated from the increase in cell numbers, were significantly (*t*-test: *p* = 0.019) lower in the warmer than in the colder treatments (9.6 ± 1.5 vs. 23.0 ± 8.0 h), with corresponding maximal growth rates (μ) of 1.77 day^-1^ and 1.10 day^-1^, respectively. Based on the appraised doubling times of the different temperature treatments, the Q_10_ value for *Glaciecola* spp. was 2.2.

Heterotrophic nanoflagellate biomass increased in synchrony with the growth of the phytoplankton bloom and with *Glaciecola* spp. abundance (**Figure 2**). In the warmer treatments, HNF biomass rose from 26 ± 8 μg C L^-1^ on day 8 to 116 ± 56 μg C L^-1^ on day 10, whereby the *Glaciecola* spp. population increased from 0.13 ± 0.08 × 10^5^ to 1.85 ± 0.50 × 10^5^ cells ml^-1^, respectively. In the colder treatments, HNF biomass increased from 22 ± 1 μg C L^-1^ on day 8 to 54 ± 9 μg C L^-1^ on day 13, whereby the *Glaciecola* spp. population increased from 0.16 ± 0.04 × 10^5^ to 0.77 ± 0.31 × 10^5^ cells ml^-1^. Hence, the maximum calculated grazing pressure occurred during the highest *Glaciecola* spp. abundance in the warmer treatments and shortly before the *Glaciecola* spp. peak in the colder treatments.

**FIGURE 3:**
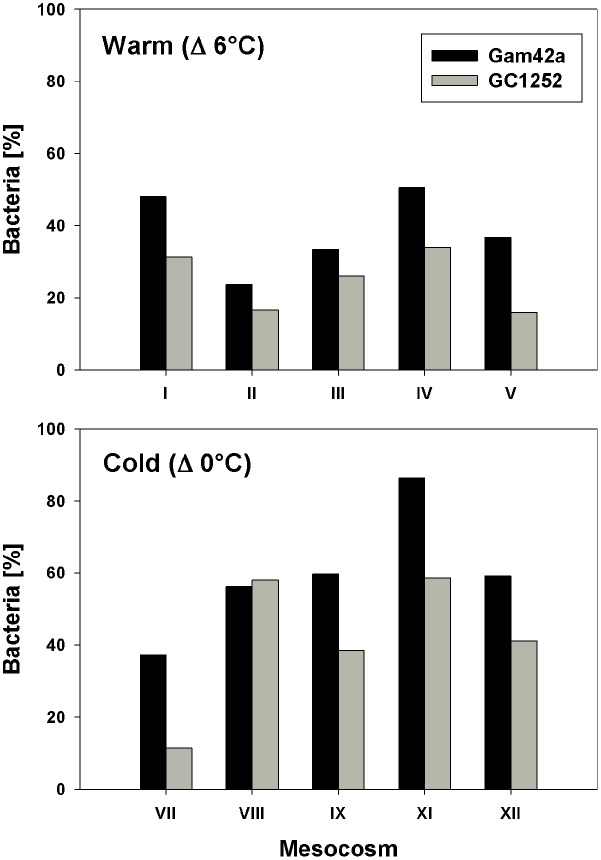
**Proportion of *Gammaproteobacteria* (Gam42a) and *Glaciecola* (GC1252) with respect to total Bacteria (EUB I-III) during the phytoplankton bloom peak in response to warm and low temperatures and for all single mesocosms (warm = day 13 of the experiment; cold = day 16 of the experiment)**.

During the phytoplankton bloom peak, the average number of total free-living bacterial cells was clearly higher in the warmer (2.15 ± 0.08 × 10^6^, at day 13) than in the colder (0.64 ± 0.10 × 10^6^, at day 17) mesocosms, with 1.74 ± 0.49 × 10^6^ cells (85 ± 7%) and 0.36 ± 0.05 × 10^6^ cells (60 ± 10%) attributable to the *Bacteria* probe (EUB I-III), respectively. At the same time, *Gammaproteobacteria* predominated, with a relative abundance in the warmer and colder treatments of 32 ± 9 and 33 ± 6% of total free-living cells, respectively. Within this bacterial group, *Glaciecola* spp. accounted for 65 ± 13 and 69 ± 30% of total *Gammaproteobacteria* in the warmer and colder treatments, respectively (**Figures 2** and **3**). The maximum abundances of *Glaciecola* spp. were detected in the warmer treatments at the phytoplankton peak (4.20 ± 1.73 × 10^5^ cells, day 13) and in the colder treatments 1 day earlier (1.45 ± 0.82 × 10^5^ cells, day 16). Although *Glaciecola* spp. achieved higher growth rates at warmer temperatures, its proportions at the phytoplankton peak were similar such that the difference between the two temperature scenarios was not significant (*t*-test: *p* = 0.578).

Micro-CARD-FISH analysis revealed that during the phytoplankton bloom peak, 65% of *Bacteria* (EUB I-III) showed activity, based on their incorporation of [^3^H]-leucine (data not shown). However, no [^3^H]-leucine uptake was detected for *Glaciecola* spp. despite the fact that at least 200 cells that hybridized with probe GC1252 were microscopically examined. The inability of *Glaciecola* spp. to incorporate [^3^H]-leucine implies that cell production by these bacteria was not included in the overall calculations of bacterial production. The free-living life style of *Glaciecola* spp. during the phytoplankton bloom peak was confirmed by micro CARD-FISH analysis with non-pre-filtered water, as none of the cells attached to algae or detritus particles.

According to the measured cell sizes of *Glaciecola* spp., the mean cell volume of these bacteria was ∼40% higher than the average bacterial cell volume. *Glaciecola* spp. reached its highest biomass in the warmer treatments on day 10 and in the colder treatments at the phytoplankton peak, which occurred on day 16 (34 ± 12 and 29 ± 19% of total bacterial biomass, respectively). During the proliferation phase of the phytoplankton bloom, total primary production was clearly lower in the warmer than in the colder treatment. By contrast, bacterial production was strongly enhanced at the warmer temperature, as evidenced by the ∼60% higher measured bacterial production (BPm) and the nearly two-fold higher estimated production rate of *Glaciecola* spp. (BP_GC_) (**Table 2**). This enhanced bacterial activity under warmer conditions in combination with the reduced autotrophic production resulted in clearly higher ratios of BPm and BP_GC_ to PP (**Table 2**).

**Table 2:** Primary production (PP), measured bacterial production (BP_m_), estimated bacterial production of *Glaciecola* spp. (BP_GC_), and the resulting total bacterial production (BP_t_) for the two mesocosm temperature regimes (Δ0°C and Δ6°C), during the proliferation phase of the phytoplankton bloom (days 8–13 for Δ°6C; days 10–16 for Δ°0C) and the phytoplankton bloom peak.

	PP	BP_m_	BP_GC_	BP_T_	Ratio [BP_GC_/BP_m_]	Ratio [BP_GC_/PP]	Ratio [BP_T_/PP]
Cold (bloom)	1528 ± 566	118 ± 6	33 ± 4	151 ± 8	0.28 ± 0.03	0.02 ± 0.01	0.11 ± 0.05
Cold (bloom peak)	322 ± 123	25 ± 5	9 ± 4	33 ± 9	0.34 ± 0.14	0.03 ± 0.02	0.13 ± 0.07
Warm (bloom)	1038 ± 241	193 ± 31	63 ± 30	256 ± 60	0.32 ± 0.01	0.06 ± 0.02	0.25 ± 0.03
Warm (bloom peak)	267 ± 78	43 ± 8	13 ± 6	59 ± 13	0.30 ± 0.10	0.05 ± 0.03	0.23 ± 0.09


*The resulting ratios of BP to PP were calculated as indicators of carbon flow from phyto- to bacterioplankton.*

The collapse of the phytoplankton spring bloom was initiated by nutrient depletion (Biermann et al., 2014). This was paralleled by a sharp decline in the abundance of *Glaciecola* spp. within 2 days, to 0.38 ± 0.67 × 10^5^ cell ml^-1^ (2.7 ± 4.9%) and 0.30 ± 0.20 × 10^5^ cell ml^-1^ (4.5 ± 3.0%) on day 18 in the warmer and colder treatments, respectively. In both, *Glaciecola* abundances remained low until the end of the experiment (**Figure 2**). Interestingly, the dynamics of *Glaciecola* spp. were much more pronounced than those of total *Gammaproteobacteria*, whose abundance remained high or even increased further (in the colder) until the end of the experiment. The pattern was indicative of a succession within this bacterial group during the degradation of the phytoplankton bloom.

The occurrence of *Glaciecola* spp. was also determined in spring bloom mesocosm experiments performed in 2006 (Sommer et al., 2007; Hoppe et al., 2008; Wohlers et al., 2009) by again applying probe GC1252 to samples filtered and stored in the same way as in the above-described experiment (**Table 3**). Thus, pre-bloom, bloom, and post-bloom samples from mesocosms in which water temperatures were maintained at Δ0°C +Δ2°C +Δ4°C +Δ6°C were examined for *Glaciecola* spp. The results confirmed the high proportion of *Glaciecola* spp. in all treatments, as evidenced by abundances of 2–12% (5.0 ± 3.5%) of all bacterial cells close to the phytoplankton bloom peak and independent of the water temperature (**Table 3**).

**Table 3:** Maximum proportion of *Glaciecola* spp. with respect to total free-living cells, water temperature, and Chl *a* concentration in two different AQUASHIFT experiments and in the Baltic Sea during March 2009 at two stations where an early phytoplankton bloom was detected.

Sample	Temperature	Chl *a*	Max proportion
	[°C]	[μg l^-1^]	[%]
AQUASHIFT mesocosms 2006	2.4	52	10.6
	4.4	56	3.6
	6.4	39	3.2
	8.4	48	11.5
			
AQUASHIFT mesocosms 2008	2.4	42.5	34.9
	8.4	34.5	36.4
			
Baltic Sea, March 2009	2.4	∼0.5	0.3
	3.2	∼ 9	0.1


*For the Baltic Sea, Chl *a* was estimated from phytoplankton biomass based on a Chl *a*:phytoplankton carbon ratio of 1:40 (Cloern et al., 1995).*

To examine the abundance of *Glaciecola* spp. under comparable *in situ* conditions in the Baltic Sea, surface water samples were collected in brackish areas from the Gulf of Finland to the southern Baltic Sea during early spring (March), when water temperatures are low (-0.3 to 3.1°C). In the northern and central Baltic Sea, phytoplankton biomass was very low (<50 μg C L^-1^) and *Glaciecola* spp. was not detected. At the more southern stations, sites of an ongoing diatom-dominated phytoplankton bloom, the maximum phytoplankton biomass detected was 347 μg C L^-1^ and *Skeletonema costatum* was the dominant phytoplankton taxa (von Scheibner et al., in preparation). At these southern stations, *Glaciecola* spp. was detectable but only in low numbers, accounting for 0.01–0.3% of the total DAPI-stained prokaryotic cells.

## Discussion

The successful enumeration of *Glaciecola* cells with the CARD-FISH probe GC1252 revealed a positive correlation between *Glaciecola* spp. abundance and Chl *a* during the exponential growth phase of the phytoplankton spring blooms in all treatments. *Glaciecola* spp. represented a large part of the bacterial community under both colder and warmer temperatures and dominated the free-living bacterial community. Probe GC1252 was primarily designed to detect a dominant OTU belonging to *Glaciecola*, as among the tested probes the GC1252 sequence showed the highest specificity within the smallest phylogenetic range in combination with the lowest outgroup hits (**Supplementary Table S2**). However, GC1252 not only targets the dominant *Glaciecola* OTU (up to 55% of total clones) but also another *Glaciecola* OTU present within the clone libraries at low abundance (∼1% of total clones). Nonetheless, the highly abundant *Glaciecola* species probably largely accounts for the reported results.

A temperature increase of 6°C enhanced the growth of *Glaciecola* spp., but the general growth pattern at warmer and colder temperatures were remarkably similar. It consisted of a rapid buildup of the populations that paralleled phytoplankton bloom development and the rapid collapse of the *Glaciecola* population after phytoplankton numbers had peaked (**Figure 2**). Adaptation by the genus *Glaciecola* to low temperatures and cold environments is suggested by a number of studies. A recent metagenomic analysis of the genus *Glaciecola* identified several cold-adapted mechanisms in the pan-genome that would allow the survival and growth of this genus at low temperatures (Qin et al., 2014). Although cold environments (e.g., in polar regions) seem to be the favored habitat of *Glaciecola* (Bowman et al., 1998; Nichols et al., 1999; van Trappen et al., 2004; Zhang et al., 2006; Prabagaran et al., 2007), its members have also been found in Mediterranean coastal waters (Alonso-Sáez et al., 2007) and during a phytoplankton spring bloom in the North Sea (Teeling et al., 2012).

*Glaciecola*, and the family *Alteromonadaceae* as a whole, is generally not an abundant member of bacterioplankton communities, but their abundance can rapidly increase following the input of labile phytoplankton-derived DOC (Eilers et al., 2000; Grossart et al., 2005; Mou et al., 2008; McCarren et al., 2010; Gómez-Consarnau et al., 2012). This is probably due to an up-regulation of metabolic genes associated with DOM utilization at optimal substrate concentrations (Tada et al., 2011). This conclusion is also supported by a metatranscriptomic study, in which, after the addition of diatom-derived DOM, *Glaciecola* sp. (HTCC2999) dominated carbohydrate metabolism pathways (Beier et al., 2015). Furthermore, growth experiments with specific DOC compounds revealed the generalist nature of some phylotypes in the genus *Glaciecola*, based on their ability to respond to a broad range of carbon compounds (e.g., *Glaciecola pallidula*), and the specialist nature of others, as demonstrated by their use of a highly restricted number of carbon compounds (e.g., pyruvate) (Gómez-Consarnau et al., 2012). Although the DOM composition in our mesocosms was not determined, the two dominant diatom species, *Skeletonema costatum* and *Thalassiosira rotula*, most likely provided exudated DOC compounds during their active growth phase and thereby effectively generated an ecological niche for *Glaciecola*. In a previous mesocosm experiment with a similar experimental setup and phytoplankton composition, DOC dynamics and composition were itemized in detail (Engel et al., 2011). In that study, large increases in the percentages of deoxysugars (mainly rhamnose and fucose) were registered before the peak of the bloom and decreased thereafter (Engel et al., 2011). This pattern is remarkably similar to that of the growth dynamics of *Glaciecola* and indicative of specific DOC-compounds as regulating factors for these bacteria. A limited labile organic carbon supply does not account for the sharp decline in *Glaciecola* spp. after the phytoplankton peak, since during this phase of the bloom both total DOC and mono-/polysaccharide concentrations strongly increased (Biermann et al., 2014). Rather, the changes in the DOC composition from the active phytoplankton growth phase to the nutrient-limited phytoplankton decline were likely to have regulated the growth of *Glaciecola* spp. in our experiment and suggest an ecological niche of this group. This conclusion is supported by batch-culture experiments in which, among the free-living bacterial community, the presence of *Gammaproteobacteria* (*Glaciecola* and *Pseudoalteromonas*) was associated exclusively with the growth phases of the diatoms *Thalassiosira rotula* and *Skeletonema costatum* (Sapp et al., 2007).

In addition to the assumed changes in DOC composition after the phytoplankton bloom peak, reinforced grazing pressure exerted by small protists may have contributed to the decline in *Glaciecola* spp. abundance (**Figures 2E,F**). *Gammaproteobacteria*, and especially *Alteromonas*, are particularly vulnerable to protist grazing, as indicated in previous studies that demonstrated selective flagellate grazing on these large, metabolically active bacteria (Beardsley et al., 2003; Worden et al., 2006; Allers et al., 2007). The high rate of phytoplankton-derived DOC utilization by *Glaciecola* and subsequent grazing by bacterivores may constitute a characteristic link in the microbial food web during phytoplankton blooms.

Considering the above-average cell size of *Glaciecola* spp., its estimated biomass was about one-third of the total bacterial biomass, indicating an enormous impact of one narrow bacterial clade (or even one species) on carbon processing during the proliferative stage of the phytoplankton bloom. Interestingly, given that *Glaciecola* spp. proved to be incapable of [^3^H]-leucine uptake, we no doubt highly underestimated bacterial production (von Scheibner et al., 2014). The inability of certain bacterial taxa to incorporate leucine or thymidine, both of which are used to assess bacterial productivity, is not unusual (Salcher et al., 2013). For this experiment, we estimate that total BP was at least 30% higher during the bloom proliferation phase than during other phases (**Table 2**) since *Glaciecola* spp. was not covered by the leucine incorporation approach.

*In situ* growth rates were calculated based on the net increase in *Glaciecola* spp. abundance and therefore can be considered as lower-end estimates, because mortality due to grazing or viral lysis was not considered. Further, a more frequent sampling interval might have provided slightly higher growth rate estimates. However, the maximum calculated growth rates were similar to those determined in earlier batch culture experiments at low temperatures. For example, the generation time of the isolate *Glaciecola punicea* was ∼13 h at 8°C and ∼26 h at 2°C (Nichols et al., 1999). A comparable net growth rate (μ ≈ 2.3 day^-1^) was determined for the family *Alteromonadaceae* in incubation experiments, and it increased after the reduction of predators (μ ≈ 4.0 day^-1^) and viruses (μ ≈ 5.8 day^-1^) (Ferrera et al., 2011). The high growth rates at cold temperatures may explain the ability of *Alteromonadaceae* to rapidly proliferate such that it dominates microbial communities exposed to a substrate pulse (Eilers et al., 2000; Beardsley et al., 2003; Alonso-Sáez et al., 2007; Mou et al., 2008). Substrate pulses, such as occur during phytoplankton blooms, can promote the expansion of specific bacterial populations (Pernthaler and Amann, 2005), which in turn can briefly but strongly impact carbon processing and transfer to higher trophic levels and thereby disrupt the general seasonal bacterial succession (Lindh et al., 2015).

The detection of a substantial abundance of *Glaciecola* spp. also in a previous mesocosm experiment that included a diatom bloom at low water temperature (**Table 3**) suggests that our results exemplify a general pattern. The relative low abundance of *Glaciecola* spp. in the southern Baltic Sea during an early spring bloom is a deviation from this pattern (**Table 3**) but might have resulted from the relatively low phytoplankton biomass or differences in phytoplankton community composition. Thus, only intensive and detailed studies can reveal natural seasonal bacterial succession involving transiently expanding bacterial taxa. For example, Teeling et al. (2012) carried out a detailed investigation of the bacterioplankton response to a diatom bloom in the North Sea. Their results suggested that algal substrate availability provides a series of ecological niches in which specialized populations, such as the *Gammaproteobacteria Glaciecola* spp. and *Reinekea* spp., can increase (Teeling et al., 2012).

Since the original description of the genus *Glaciecola* as comprising gram-negative, aerobic, psychrophilic slightly halophilic bacteria (Bowman et al., 1998), there have been many extensions and modifications (van Trappen et al., 2004; Baik et al., 2006; Zhang et al., 2006; Shivaji and Reddy, 2014). However, most species seem to be psychrophilic, including those responsible for the results of this study, which achieved high *in situ* net growth rates at a low temperature of ∼2°C. This is in accordance with the lack of a significant temperature effects on the relative abundance of *Glaciecola* spp. Also the relatively low Q_10_ value of 2.2 is within the normal range of temperature-accelerated bacterial growth (Kirchman et al., 2009). Remarkably, despite higher grazing pressure from HNF, the growth rate of *Glaciecola* spp. at the low temperature was high (∼1.10 day^-1^), in contrast to that of the total bacterial community (∼0.4 day^-1^).

Yet, besides direct temperature effects, the growth dynamic of *Glaciecola* spp. determined in this experiment could also have been influenced by biotic interactions within a complex food-web as well as by differences in phytoplankton community composition or grazer abundance (Lewandowska and Sommer, 2010; Aberle et al., 2012; Winder et al., 2012). Overall, the coincidence of the high growth rate and abundance of *Glaciecola* spp. with the onset of the phytoplankton bloom indicates a tight coupling between blooming diatoms and *Glaciecola* spp. Moreover, this relationship provides evidence of a key role of cold-adapted *Glaciecola* spp. in the processing of phytoplankton-derived DOC during the proliferation of phytoplankton at low water temperatures. Increasing the experimental temperature did not substantially change this relationship, as *Glaciecola* spp. remained the dominant bacterial taxa also after warming, although other bacterial groups gained in importance as well (von Scheibner et al., 2014). Whether *Glaciecola* is replaced by other taxa at temperatures higher than that tested here and how this dynamic is regulated by the interplay of substrate composition, grazing, and viral lysis remain to be determined. It will also be interesting to compare our results with those obtained in other marine environments (e.g., those with higher salinity), where different bacterial taxa dominate the respective carbon flow during early phytoplankton blooms.

## Author Contributions

MvS, US, KJ designed the experiment. MvS performed the sampling and analysis. MvS, US, KJ wrote the manuscript.

## Conflict of Interest Statement

The authors declare that the research was conducted in the absence of any commercial or financial relationships that could be construed as a potential conflict of interest.
