# Supplementary material for: Tight Coupling of Glaciecola spp. and Diatoms during Cold-Water Phytoplankton Spring Blooms
Source: Front Microbiol. 2017 Jan 19;8:27. doi: 10.3389/fmicb.2017.00027 (PMC5243806; doi:10.3389/fmicb.2017.00027)

Supplementary information

"Tight coupling of Glaciecola spp. and diatoms during cold-water phytoplankton spring blooms"

by Markus von Scheibner, Ulrich Sommer, Klaus Jürgens

Table S1: Comparison of the probe sequence GC1252 with the target sequences of several species within the *Glaciecola* family and three outsider sequences within the *Gammaproteobacteria*.

Bacterial strain	Orientation	Sequence	No. Mismatches
Probe GC1252	3′ -> 5′	TCC CTA CGT TTG ACC ACT GTC A	
Target sequence	5′ -> 3′	AGG GAU GCA AAC UGG UGA CAG U	
Clone 1 (warm)	5′ -> 3′	*** *** *** *** *** *** *** *	(0 Mismatches)
Clone 175 (cold)	5′ -> 3′	*** *** *** *** *** *** *** *	(0 Mismatches)
Glaciecola pallidula	5′ -> 3′	*** *** *** *** *** *** *** *	(0 Mismatches)
Glaciecola punicea	5′ -> 3′	*** *C* *** *** *A* *** U** *	(3 Mismatches)
Glaciecola polaris	5′ -> 3′	*** **A *** *** *U* C** G** *	(4 Mismatches)
Glaciecola mesophila	5′ -> 3′	*** **A *** *** *U* C** G** *	(4 Mismatches)
Glaciecola psychrophila	5′ -> 3′	*** **A *** *** CU* C** GG* *	(6 Mismatches)
Glaciecola sp. HTCC 2999	5′ -> 3′	*** *** **G *** CU* C** AG* *	(6 Mismatches)
Alteromonas macleodii	5′ -> 3′	*** **A *** *GA CA* *** UGU G	(9 Mismatches)
Colwellia phychrerythraea 34H	5′ -> 3′	*** *CA *** *UA CC* C** GGU G	(11 Mismatches)
Escherichia coli	5′ -> 3′	**A **A **G *C* *C* C** G** C	(8 Mismatches)

Table S2: Table of the matched organisms detected with probe GC1252 and confirmed using SILVA TestProbe 3.0 (http://www.arb-silva.de/search/testprobe/) with SILVA release 123 NR and the sequence collection REFNR (effective Jan. 2016).

Accession no.	Path	Organism name
KC899218	Bacteria;Actinobacteria;Actinobacteria;Micrococcales;Microbacteriaceae;Candidatus Aquiluna;	Uncultured Micrococcineae bacterium
AY697917	Bacteria;Proteobacteria;Alphaproteobacteria;Rhodobacterales;Rhodobacteraceae;uncultured;	Uncultured rhodobacteraceae bacterium
AUAV01000034	Bacteria;Proteobacteria;Gammaproteobacteria;Alteromonadales;Alteromonadaceae;Glaciecola;	Glaciecola pallidula dsm 14239 = acam 615
AY697880	Bacteria;Proteobacteria;Gammaproteobacteria;Alteromonadales;Alteromonadaceae;Glaciecola;	Uncultured glaciecola sp.
AY697894	Bacteria;Proteobacteria;Gammaproteobacteria;Alteromonadales;Alteromonadaceae;Glaciecola;	Uncultured glaciecola sp.
AY697921	Bacteria;Proteobacteria;Gammaproteobacteria;Alteromonadales;Alteromonadaceae;Glaciecola;	Uncultured glaciecola sp.
AY787042	Bacteria;Proteobacteria;Gammaproteobacteria;Alteromonadales;Alteromonadaceae;Glaciecola;	Glaciecola nitratireducens
AY794092	Bacteria;Proteobacteria;Gammaproteobacteria;Alteromonadales;Alteromonadaceae;Glaciecola;	Uncultured glaciecola sp.
AY794116	Bacteria;Proteobacteria;Gammaproteobacteria;Alteromonadales;Alteromonadaceae;Glaciecola;	Uncultured glaciecola sp.
AY794130	Bacteria;Proteobacteria;Gammaproteobacteria;Alteromonadales;Alteromonadaceae;Glaciecola;	Uncultured glaciecola sp.
AY794156	Bacteria;Proteobacteria;Gammaproteobacteria;Alteromonadales;Alteromonadaceae;Glaciecola;	Uncultured alteromonas sp.
BAEQ01000059	Bacteria;Proteobacteria;Gammaproteobacteria;Alteromonadales;Alteromonadaceae;Glaciecola;	Glaciecola pallidula dsm 14239 = acam 615
CP003060	Bacteria;Proteobacteria;Gammaproteobacteria;Alteromonadales;Alteromonadaceae;Glaciecola;	Glaciecola nitratireducens fr1064
CP003060	Bacteria;Proteobacteria;Gammaproteobacteria;Alteromonadales;Alteromonadaceae;Glaciecola;	Glaciecola nitratireducens fr1064
CP003060	Bacteria;Proteobacteria;Gammaproteobacteria;Alteromonadales;Alteromonadaceae;Glaciecola;	Glaciecola nitratireducens fr1064
CP003060	Bacteria;Proteobacteria;Gammaproteobacteria;Alteromonadales;Alteromonadaceae;Glaciecola;	Glaciecola nitratireducens fr1064
EU287078	Bacteria;Proteobacteria;Gammaproteobacteria;Alteromonadales;Alteromonadaceae;Glaciecola;	Uncultured bacterium
FR746107	Bacteria;Proteobacteria;Gammaproteobacteria;Alteromonadales;Alteromonadaceae;Glaciecola;	Glaciecola pallidula
GQ349618	Bacteria;Proteobacteria;Gammaproteobacteria;Alteromonadales;Alteromonadaceae;Glaciecola;	Uncultured gamma proteobacterium
HQ203911	Bacteria;Proteobacteria;Gammaproteobacteria;Alteromonadales;Alteromonadaceae;Glaciecola;	Uncultured bacterium
HQ225195	Bacteria;Proteobacteria;Gammaproteobacteria;Alteromonadales;Alteromonadaceae;Glaciecola;	Uncultured bacterium
HQ225314	Bacteria;Proteobacteria;Gammaproteobacteria;Alteromonadales;Alteromonadaceae;Glaciecola;	Uncultured bacterium
HQ875493	Bacteria;Proteobacteria;Gammaproteobacteria;Alteromonadales;Alteromonadaceae;Glaciecola;	Glaciecola sp. Za3-19
HQ875494	Bacteria;Proteobacteria;Gammaproteobacteria;Alteromonadales;Alteromonadaceae;Glaciecola;	Glaciecola sp. Za3-36-1

JX015904	Bacteria;Proteobacteria;Gammaproteobacteria;Alteromonadales;Alteromonadaceae;Glaciecola;	Uncultured bacterium
KC139305	Bacteria;Proteobacteria;Gammaproteobacteria;Alteromonadales;Alteromonadaceae;Glaciecola;	Uncultured glaciecola sp.
KC899227	Bacteria;Proteobacteria;Gammaproteobacteria;Alteromonadales;Alteromonadaceae;Glaciecola;	Uncultured glaciecola sp.
KC899232	Bacteria;Proteobacteria;Gammaproteobacteria;Alteromonadales;Alteromonadaceae;Glaciecola;	Uncultured glaciecola sp.
KC899236	Bacteria;Proteobacteria;Gammaproteobacteria;Alteromonadales;Alteromonadaceae;Glaciecola;	Uncultured glaciecola sp.
KC899248	Bacteria;Proteobacteria;Gammaproteobacteria;Alteromonadales;Alteromonadaceae;Glaciecola;	Uncultured glaciecola sp.
U73723	Bacteria;Proteobacteria;Gammaproteobacteria;Alteromonadales;Alteromonadaceae;Glaciecola;	Gas vacuolate str. 206
U85854	Bacteria;Proteobacteria;Gammaproteobacteria;Alteromonadales;Alteromonadaceae;Glaciecola;	Glaciecola pallidula
AY165576	Bacteria;Proteobacteria;Gammaproteobacteria;Alteromonadales;Alteromonadaceae;Paraglaciecola;	Uncultured antarctic sea ice bacterium
FR683301	Bacteria;Proteobacteria;Gammaproteobacteria;Oceanospirillales;Oceanospirillaceae;Balneatrix;	Uncultured marine bacterium
KC899234	Bacteria;Proteobacteria;Gammaproteobacteria;Pseudomonadales;Moraxellaceae;Perlucidibaca;	Uncultured gamma proteobacterium

Fig. S1:

Microscopy images of the free-living bacterial population (3.0-µm, prefiltered) during the peak of the phytoplankton spring bloom, including 4',6-diamidino-2-phenylindole (DAPI)-stained cells in blue (left) and GC1252-stained cells in green (right).

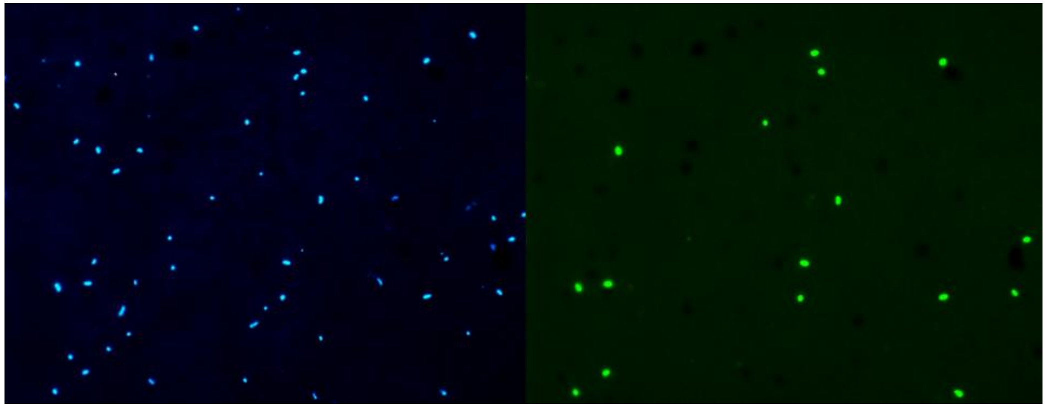

Supplement: Supplementary file 1 [file Presentation_1.PDF]
